# A Case of Umbilical Cord Prolapse With Intact Membranes Managed Successfully With Conservative Measures

**DOI:** 10.7759/cureus.29870

**Published:** 2022-10-03

**Authors:** Carmen A Cueto, Emi Komatsu, Samantha G Lee, Brian Gordon

**Affiliations:** 1 Department of Obstetrics and Gynecology, Los Angeles County University of Southern California Medical Center, Los Angeles, USA

**Keywords:** ultrasound utilization, cord prolapse identification, trendelenburg position, intact membranes, cord prolapse management

## Abstract

Umbilical cord prolapse with ruptured membranes is an obstetric emergency with management consisting of delivery via emergent cesarean delivery. If the umbilical cord prolapses beyond the internal os with intact membranes, there is an opportunity to intervene and reduce the risk of fetal morbidity and mortality. A healthy 30-year-old, gravida 1 para 0 was incidentally found to have a short cervical length at 25 weeks five days on routine anatomy ultrasound evaluation. On evaluation via ultrasound by the maternal-fetal medicine service, the umbilical cord was noted to be prolapsing through the cervix with membranes intact. The cord prolapse with intact membranes resolved after placing the patient in the Trendelenburg position and nifedipine was administered for tocolysis given the uterus was noted to be contracting. For the remainder of the pregnancy, the patient underwent close follow-up and serial ultrasound scans with confirmation of the fetal head as the presenting part. The patient ultimately delivered vaginally at term. Cord prolapse with intact membranes, when identified via ultrasound, can be managed conservatively via Trendelenburg positioning and tocolysis to avoid premature cesarean delivery.

## Introduction

Umbilical cord prolapse is an obstetric emergency that occurs in 1-6 per 1,000 pregnancies [1,2]. Cord prolapse with intact membranes refers to the cord prolapsing beyond the internal and external os of the cervix with intact membranes [1]. Cord prolapse with ruptured membranes is defined as the descent of the umbilical cord below the level of the cervix in the setting of ruptured membranes, with the mainstay of treatment being urgent delivery via cesarean delivery. With ruptured membranes and fetal descent, the umbilical cord becomes at risk of being compressed between the presenting fetal part and the cervix, thereby impeding oxygen delivery to the fetus and risking neurological injury and death. When the umbilical cord is the presenting part and prolapses below the cervix with membranes intact, the risk for interruption of the oxygenation pathway to the fetus remains. In this scenario, the risk of progression to rupture of membranes increases and can lead to emergent cesarean delivery. Ultrasound evaluation in high-risk situations, including cervical insufficiency, cervical dilation, or preterm labor, can increase the likelihood of continued pregnancy and improve neonatal outcomes compared to immediate preterm delivery with subsequent morbidity [3,4]. Certain maneuvers can provide immediate relief of cord prolapse while preparing for delivery, including manually lifting the presenting fetal part, filling the maternal bladder, knee-to-chest position, and Trendelenburg position [5]. Each of these strategies can improve fetal heart rate and reduce the risk of fetal hypoxic brain injury at the time of delivery [5,6]. Distinct from cord prolapse with ruptured membranes, it is important to identify cord prolapse with intact membranes to proactively manage and avoid membrane rupture and delivery. Early identification of cord prolapse with intact membranes can avoid the significant fetal morbidity and mortality associated with cord prolapse with ruptured membranes [1,2]. Whether these same maneuvers applied to relieve cord prolapse with ruptured membranes also provide benefit and/or affect outcomes in the setting of cord prolapse with intact membranes is not well-documented. The objective of this case report is to highlight the use of conservative measures in relieving cord prolapse in a patient with intact membranes.

## Case presentation

**Figure 1:**
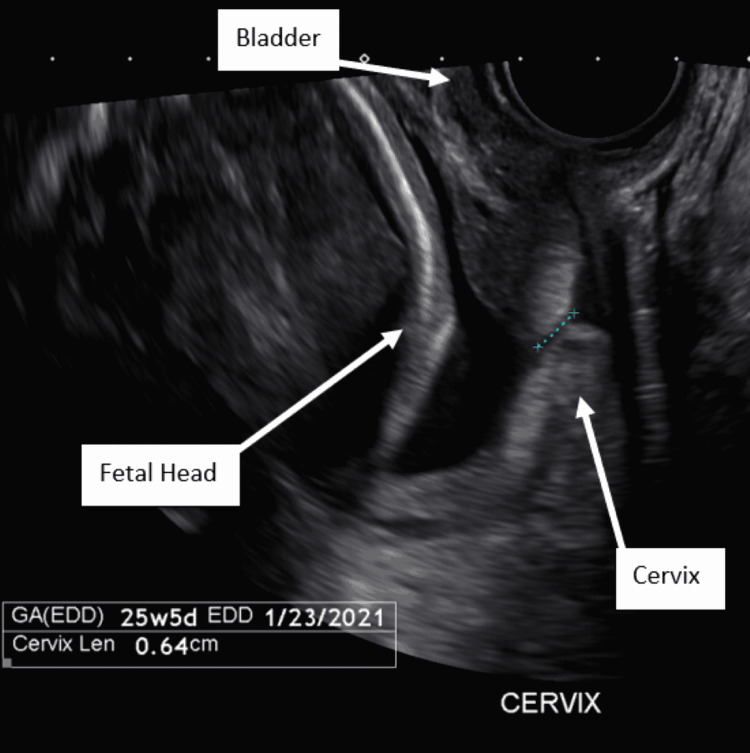
Transvaginal ultrasound cervical length at the time of presentation (0.64 cm).

A 30-year-old, gravida 1 para 0, patient presented at 25 weeks five days gestation after a referral from radiology given an incidental finding of a 0.64 cm cervical length on a routine anatomy scan (Figure 1). The patient was sent to the obstetrics triage unit for further evaluation. She reported intermittent cramping pain and was found to have contractions on the tocodynamometer. Given concern for preterm labor in the setting of a short cervical length, the patient was admitted for corticosteroids for fetal lung maturity and continuous fetal heart rate monitoring.

**Figure 2:**
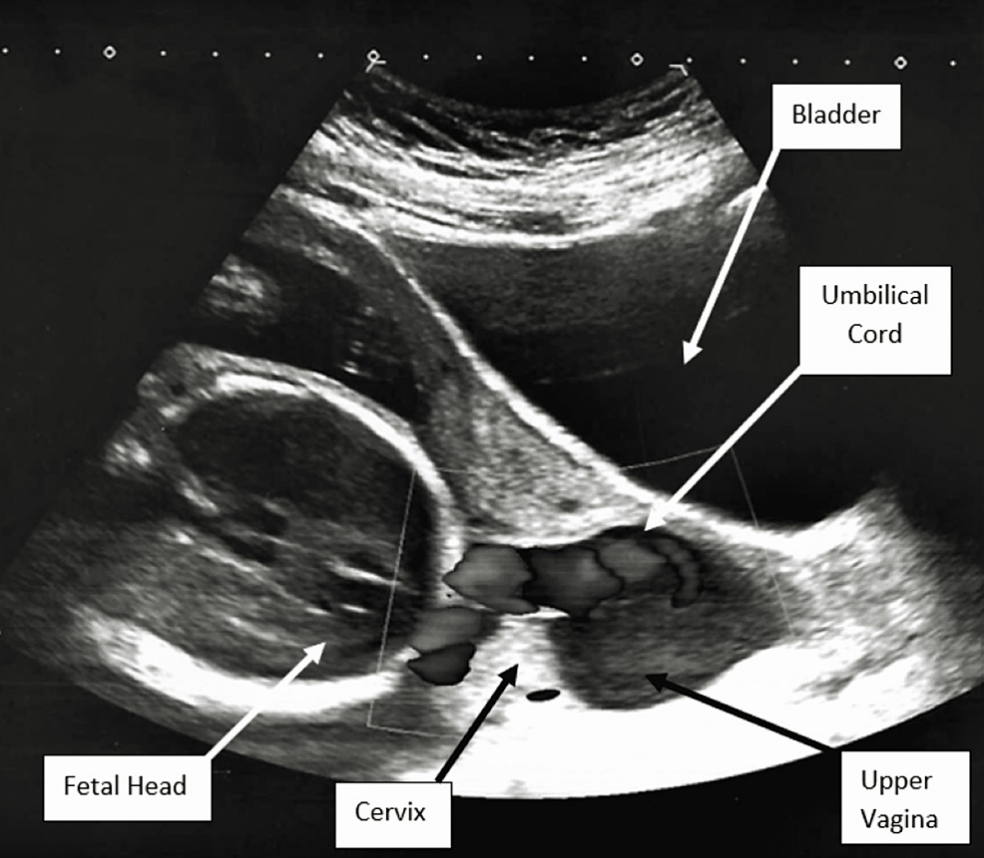
Transabdominal ultrasound identification of umbilical cord prolapse with intact membranes.

On hospital day one, the patient was scanned by the maternal-fetal medicine service given the concern for a shortened cervix. On transabdominal ultrasound, the cervical length appeared consistent with the 6 mm length seen on prior imaging, and the diagnosis of cord prolapse with intact membranes was made as the umbilical cord was noted to be prolapsing into the upper vagina (Figure 2). Amniotic membranes were visually intact with a maximum vertical pocket of 4.56 cm. There was no leakage of fluid appreciated on the sterile speculum examination, and documentation noted that the cervix was visually closed with the amniotic bag seen in the vagina. The patient was placed in the Trendelenburg position and counseled on the ultrasound and examination findings. Cesarean delivery consents were reviewed and signed given she desired full intervention should an emergency arise. In anticipation of preterm delivery, magnesium sulfate was started for neuroprotection and the first dose of intramuscular betamethasone was administered. Nifedipine 20 mg every six hours was started for tocolysis through the steroid window given the patient continued to experience painful contractions. The fetal heart rate tracing remained category one throughout. After two hours of Trendelenburg positioning, resolution of the cord prolapse was noted on repeat transabdominal ultrasound. The repeat ultrasound findings revealed a well-applied fetal head with confirmation of no cord presentation on color Doppler. A digital cervical examination was done at this time noting the patient to be 1 cm dilated, 90% effaced, and -2 fetal station. On hospital day two, the second dose of intramuscular betamethasone was given and nifedipine was continued through the steroid window.

The patient remained admitted for close monitoring given intermittent contractions, short cervix, and early gestational age for eight additional days. A urinalysis and wet mount were negative for urinary tract infection and vaginitis, thus ruling out other possible causes for uterine activity. Subsequent daily ultrasound continued to confirm resolution of cord prolapse, with the cervical exam stable at 1 cm dilated, 90% effaced, and -2 station. A repeat speculum examination was conducted and confirmed no prolapsing membranes at the cervical os. Sequential compression devices were used for venous thromboembolism prophylaxis in the setting of a prolonged hospital stay. The patient was given the option of continued inpatient management until delivery; however, she elected to be discharged home at 26 weeks and six days with close follow-up and was given strict return precautions. Subsequent ultrasounds continued to demonstrate a stable shortened cervix at 6 mm without evidence of cord prolapse. She received routine prenatal care with serial ultrasounds for the remainder of her pregnancy and presented at 37 weeks four days with spontaneous rupture of membranes in cephalic presentation and had an uncomplicated vaginal delivery.

## Discussion

To reduce neonatal morbidity and mortality, management of cord prolapse with rupture of membranes is emergent cesarean delivery [1,3-5]. For this reason, when discussing cord prolapse, it is important to recognize that membrane status greatly impacts management. Intact membranes provide the opportunity to conservatively manage and avoid emergent delivery when cord prolapse is identified. If this case had been managed as cord prolapse with ruptured membranes, or if the management had not been successful, preterm cesarean delivery would have occurred. Risk factors for umbilical cord prolapse include malpresentation, multiparity, low birth weight, polyhydramnios, velamentous cord insertion [4], and, in this case, the risk for preterm delivery and short cervical length. Being aware of these risk factors allows for the utilization of ultrasound for early identification of cord prolapse with intact membranes and conservative management measures to avoid premature rupture of membranes and emergent delivery [3]. Applying a color Doppler while checking a patient’s cervical length can readily identify the umbilical cord within the cervical canal with intact membranes. Upon confirming the diagnosis of cord presentation, placing the patient in the Trendelenburg position successfully reduced the prolapse by allowing gravity and maternal repositioning to restore the potential space the umbilical cord was previously occupying. Restoring this space ultimately allows for the fetal head to descend into the pelvis and reduces the risk of recurrent cord prolapse.

Administering tocolysis contributes to the success of Trendelenburg positioning by relieving the contractions and relaxing the uterus, thus eliminating an opposing force to gravity in reducing the cord prolapse [5]. Following the management strategy described in this case report requires close clinical follow-up and serial ultrasounds to confirm presentation to reduce the risk of recurrence and ensure engagement of the presenting fetal part.

## Conclusions

This case report provides an example of the diagnosis and management of cord prolapse with intact membranes. The utilization of ultrasound imaging allows for the identification of cord prolapse with intact membranes. In high-risk patients, including those with a short cervical length, serial ultrasounds with a color Doppler may be considered for early detection of cord prolapse. We demonstrate how conservative management through the use of Trendelenburg positioning and tocolysis resulted in an uncomplicated full-term vaginal delivery. This presents a potential alternative for similar cases to avoid progression to membrane rupture and emergent cesarean delivery.
